# Callous-Unemotional Traits in Adolescents' Daily Life: Associations with Affect and Emotional and Conduct Problems

**DOI:** 10.1007/s10802-023-01077-6

**Published:** 2023-05-30

**Authors:** Natalie Goulter, Eric M. Cooke, Yao Zheng

**Affiliations:** 1 School of Psychology, Newcastle University, Newcastle upon Tyne, UK; 2 Department of Psychology, Simon Fraser University, Burnaby, Canada; 3 Criminal Justice Program, Bowling Green State University, Bowling Green, USA; 4 Department of Psychology, University of Alberta, Edmonton, Canada

**Keywords:** Adolescence, Affect, Callous-unemotional traits, Conduct problems, Emotional problems, Intensive longitudinal data

## Abstract

Intensive longitudinal methods (e.g., daily diary) inform understanding of dynamic processes by parsing *within-person* state-like fluctuations from stable *between-person* trait-like differences. In this exploratory study, we investigated whether self-reported callous-unemotional (CU) traits (callousness, uncaring) demonstrated daily fluctuations, as well as whether daily CU traits were associated with multiple forms of daily emotional and behavioral functioning. A sample of 99 adolescents (55.8% female; *M*_age_ = 14.60 years) provided baseline information and completed a naturalistic 30-day diary reporting on CU traits, positive and negative affect, and emotional and conduct problems in their daily lives. Dynamic structural equation modeling revealed that many CU traits items showed within-person autoregressive and cross-lagged links; however, there was substantial between-person variation in within-person fluctuations across items. At the subscale level, cross-day associations were observed between callousness and uncaring, conduct problems and uncaring, positive affect and callousness, negative affect and emotional problems, and emotional problems and negative affect. By harnessing intensive longitudinal data, our findings provide preliminary state-level evidence of CU traits, as well as functional information with regards to CU traits and emotional and behavioral problems in daily life. We consider the implications of our findings in terms of informing future CU traits intensive longitudinal evaluations.

**Supplementary Information:**

The online version contains supplementary material available at 10.1007/s10802-023-01077-6.

## Introduction

In the DSM-5, a “with Limited Prosocial Emotions” specifier was added to the Conduct Disorder diagnostic criteria to differentiate those children and adolescents who also display a callous absence of empathy and remorse, shallow or narrow affect, and deficient concern regarding performance in activities (American Psychiatric Association, 2013). This addition was based on research extending the affective dimension of the adult psychopathy personality construct downward to advance developmental theories (Frick, 2022; Frick et al., 2014). Greater knowledge on how these traits manifest earlier in development, when they may be more malleable, can inform clinical prevention and intervention efforts. In research settings, this specifier is commonly referred to as callous-unemotional (CU) traits and, like psychopathy, these traits are associated with several unique behavioral, emotional, and cognitive correlates relative to other externalizing and antisocial constructs (Frick et al., 2014). However, empirical work on CU traits has relied on panel (or cross-sectional) designs that primarily investigate within-person change. Such panel designs do not sufficiently assess within-person fluctuations and have comparatively low ecological validity–two important limitations of this line of research that can be circumvented through intensive longitudinal methodologies.

### Correlates of Callous-unemotional Traits

Studies have pinpointed several distinguishing correlates of CU traits. Individuals with conduct problems and normative CU traits are often characterized by a defiant or difficult temperament with heightened levels of emotion dysregulation, negative affect, and impulsive aggression to provocation (Frick & Viding, 2009). In contrast, those with elevated conduct problems *and* CU traits do not tend to show the same types of emotional reactivity and are instead discerned by a fearless disposition reflected as emotional hypoactivity, reduced sensitivity to punishment, and often greater aggression severity (Fanti et al., 2016; McMahon et al., 2010). Despite the name, CU traits do not always confer risk for blunted emotional responsivity. For example, although differentiated on fear processing, one meta-analytic review (*k* = 20) found no differences across CU traits and control groups in processing angry, disgusted, or happy cues (Marsh & Blair, 2008). In addition to dysfunctional parenting practices, it is this fearlessness that may explain why individuals with CU traits have been less successfully served by treatment efforts (Hawes et al., 2014a; McMahon et al., 2021). Distinctions have also been made regarding the structure of CU traits, with studies having identified three (callousness, uncaring, unemotional; Ray & Frick, 2020) or two (callousness, uncaring; Hawes et al., 2014b; Zheng et al., 2021) dimensions. Callousness tends to capture deficient empathy and guilt, uncaring reflects an aloof attitude regarding others, and unemotionality is defined as impoverished emotional experiences. Regardless of the structure, CU traits discriminates an etiologically and clinically atypical group (Frick et al., 2014).

### Traits and Symptoms in Adolescence

Adolescence is a sensitive developmental period marked by profound cognitive, neurobiological, and interpersonal change (Dahl et al., 2018). In a large meta-analysis including 192 epidemiological studies, Solmi et al. (2022) found that the peak age of onset of any mental disorder was 14.5 years. Therefore, adolescence represents a prime time for understanding vulnerabilities and the development of psychopathological symptoms. Personality and psychopathology are typically considered distinct domains sometimes differentiated based on temporal stability. Specifically, some researchers have suggested that personality traits reflect long-term dispositions and that psychopathological symptoms are state-like instances of an experience or behavior (DeYoung et al., 2022). In childhood and adolescence, however, personality traits may also change over time (Soto et al., 2011). Indeed, some research has found little differences between mean-level change of several personality facets and internalizing symptoms across five 9-month follow-ups in a sample of female adolescents (Goldstein et al., 2022). Other evidence has shown that personality pathology tends to decline across adolescence (Álvarez-Tomás et al., 2019). In the CU traits literature, many panel studies have examined the long-term stability of CU traits with one review suggesting that CU traits show substantial rank-order stability and modest mean-level stability during childhood, adolescence, and into early adulthood (Frick et al., 2014). However, what cannot be ascertained from this work is whether adolescents with CU traits are consistent in their manifestation of CU traits. In other words, do CU traits fluctuate day-to-day (or moment-by-moment)?

### Intensive Longitudinal Methods

Innovations in technology (e.g., smart phones and wearables) have generated a surge in studies investigating dynamic processes on micro timescales using a range of intensive longitudinal methods (for recent reviews, see Russell & Gajos, 2020; Urben et al., 2022). Broadly, intensive longitudinal methods, such as daily diary, experience sampling, ecological momentary assessment, and other ambulatory assessment designs, are characterized by many measurement occasions over a brief observation window. These methods focus on within-person variability, such that the measured variables may show deviations or fluctuations relative to the individual mean at specific observation occasions, but the individual mean is relatively stable over time compared to developmental growth or decrease in conventional longitudinal designs. Major advantages of intensive longitudinal methods also include the capability to assess real-time experiences and memory compared to traditional longitudinal designs, which can result in retrospective and recall biases (Russell & Gajos, 2020). These methods also enhance ecological validity, such that assessments are conducted when participants are in real-world settings relative to artificial laboratory experiments. Hence, intensive longitudinal data provide a high level of temporal granularity and inform our understanding of real-life short-term within-person dynamics as opposed to long-term developmental change.

### Testing Personality and Psychopathology with Intensive Longitudinal Methods

Studies are beginning to apply intensive longitudinal designs to elucidate the dynamics of personality and psychopathology. For example, across three samples of adults, Edershile and Wright (2021) tested whether narcissism reflects a dynamic process and found variability in narcissistic states at a moment-to-moment level. Among 91 adults with a personality disorder, daily assessments of personality pathology including negative affect, detachment, impulsivity, and hostility predicted clinical characterizations of personality disorders and daily stress levels (Dotterer et al., 2019). In a sample of adolescent girls, daily negative affect, detachment, disinhibition, and psychoticism were associated with daily socio-affective processes (Kaurin et al., 2022). Such studies enhanced our understanding of state-level fluctuations in personality and psychopathology. Yet, no research to date has investigated whether CU traits also vary day-to-day or moment-to-moment. Only two intensive longitudinal studies have included a measure of CU traits using justice-involved adolescent samples; however, both studies only assessed CU traits as a total score at a single timepoint (De Ridder et al., 2016; Suter et al., 2017). Another study examined callousness in the context of interpersonal antagonism in samples of undergraduates, community adults, and psychiatric outpatients, but, similarly, callousness was only assessed at one timepoint as a predictor (Vize et al., 2022). Across these studies, higher levels of CU traits were associated with higher levels of perceived angry affect and self-reported antisocial behavior (De Ridder et al., 2016; Suter et al., 2017), and callousness was negatively associated with positive affect and empathy (Vize et al., 2022).

In line with other personality constructs (Wright & Kaurin, 2020), much of our current understanding of CU traits is founded on structural or trait-based models (Frick et al., 2014). Research on dynamic modeling at micro timescales may help further inform functional narratives. As the affective domain of psychopathy, CU traits may demonstrate meaningful within-person fluctuations. Notably, the links between CU traits and conduct problems at the daily within-person level could possibly be instantiated by their intermediate and differential links involving daily positive and negative affect. Although most research places negatively valenced emotion processes at the center of personality and psychopathology, some research has implicated positive affect in the development of aggressive behaviors (Toro et al., 2020). In addition, affect and emotion are best conceptualized as dynamic processes underpinning a range of psychopathological outcomes (Houben et al., 2015). Other theoretical accounts suggest that certain forms of personality are derived and maintained by state-level fluctuations of the traits themselves (e.g., grandiosity and vulnerability in narcissism; Edershile & Wright, 2021). It may be the case that daily fluctuations in callousness and uncaring serves to perpetuate CU traits and associated conduct problems. Overall, state-level personality may be more malleable than traits, and thus, studying phenomena at the state-level can illuminate more effective treatment targets for this typically treatment-resistant group.

### The Present Study

There is a lack of knowledge on CU traits on a micro timescale. In this exploratory work with data collected from a sample of adolescents via an intensive 30-day daily diary design, our aims were twofold. First, we aimed to explore whether CU traits (at the item level) demonstrated daily within-person fluctuations. In addition to the potential novel knowledge gained from investigating CU traits using intensive longitudinal designs, examining CU traits at the item level is congruent with recent research initiatives (e.g., Hierarchical Taxonomy of Psychopathology; Kotov et al., 2021) that emphasize fine-grained trait or symptom level analyses to advance mental health science. Second, we aimed to investigate whether daily CU traits (including callousness and uncaring) were associated with daily positive and negative affect, as well as emotional and conduct problems. To address these aims, we applied dynamic structural equation modeling (DSEM) to parse both *within-person* state-level fluctuations including autoregressive and cross-lagged associations (i.e., whether participants’ level at time *t* was significantly predicted by their level of the same or different construct, respectively, at time *t*-1) from stable *between-person* trait-like differences (Asparouhov et al., 2018; Hamaker et al., 2018). Given the exploratory nature of this study, we pose no specific a priori hypotheses.

## Method

### Participants and Procedure

Adolescents living in a western Canadian province were recruited between April 2019 and October 2020 to participate in an online study examining their psychological and behavioral adjustment (see Xu & Zheng, 2022). A total of 99 participants (12–17 years old, *M* = 14.60, *SD* = 1.76, 55.8% female, 51.5% white, 23.2% Asian, 8.1% multiracial, 4.0% Hispanic/Latinx, 2.0% Black, 6.1% Other) completed a baseline survey and at least one day of the 30-day daily diary surveys (2,108 total observations, *M* = 21.72 days, *Range* = 1–30, *SD* = 7.80). Among the adolescents, 81.1% reported living with both biological parents, 17.8% reported living with one biological parent, and 1.1% reported living with someone other than a biological parent. Most participants’ parents reported a personal annual income within the average (CAD $65,700) and median ($51,600) personal total incomes for groups of 25–54-year-olds in this Canadian province (17.2% had a personal annual income below $35,000, 9.1% were between $35,000–$45,000, 12.1% were between $45,000–$55,000, 17.2% were between $55,000–$65,000, and 38.4% had an annual income above $65,000).

The research ethics committee at the University of Alberta approved the procedure and instruments for this study. Survey instruments were developed and administered via email through RedCap. Participants were recruited through newsletters, social media, and flyers posted or circulated in the western Canadian province. All adolescents were eligible for inclusion in the study. Interested participants were asked to contact the research team and were provided with information for the study. Participants received an online baseline survey (~45 min to complete) following the obtainment of assent online. Participants’ parents provided consent online for their children to participate in this study. Daily surveys (~10 min to complete) were sent out five days after completion of the baseline survey for 30 consecutive days. The daily survey was sent out at 5 pm each day and adolescents were asked to fill out the survey before going to sleep that night. Participants received a $45 e-gift card of their choosing as compensation for their participation in the baseline and daily surveys.

### Measures

#### Callous-unemotional Traits

Daily callous-unemotional traits were assessed with a shortened 12-item version of the 24-item Inventory of Callous Unemotional Traits (ICU; Hawes et al., 2014b; Zheng et al., 2021; see Table 1). Each item is scored on a 4-point Likert scale (0 = *not at all true*, 1 = *somewhat true*, 2 = *mostly true*, 3 = *definitely true*) in response to the stem “indicate how well the following statements described you today.” This ICU scale includes two subscales: callousness (6 items; ordinal ω_w_ = 0.48, ordinal ω_b_ = 0.91) and uncaring (5 items; ordinal ω_w_ = 0.71, ordinal ω_b_ = 0.91). Items that needed to be reverse-coded were done so prior to the analysis. Multilevel Confirmatory Factor Analyses (MLCFA) with the weighted least squares means and variances adjusted estimator demonstrated that the 2-factor model provided satisfactory and better model fit at within- and between-levels (CFI = 0.97, TLI = 0.97, RMSEA = 0.02, SRMR_within(w)_ = 0.08, SRMR_between(b)_ = 0.04), compared to the 1-factor model at both levels (CFI = 0.87, TLI = 0.83, RMSEA = 0.04, SRMR_w_ = 0.15, SRMR_b_ = 0.07). The estimated averaged standardized factor loading for the callousness factor was 0.60 and 0.84 at within- and between-levels, and for the uncaring factor was 0.70 and 0.80 at within- and between-levels. MLCFA using the maximum likelihood estimator with robust standard errors (MLR) demonstrated that the 2-factor model at within- and between-levels provided better model fit (Δ scaling correction = 3.68, Δχ^2^ = 163.03, Δdfs = 2.00, *p* < 0.001) compared to the 1-factor model at both levels. For the first aim, all 12-items were used. However, in line with several studies that have found that item 3 (*does not show emotions*), the only item left from the original unemotional subscale, exhibits low factor loading (Colins et al., 2016; Zheng et al., 2021), this item was not included in calculations of subscales for the second aim.

**Table 1:** Callous-Unemotional Traits Items and Subscales

*Item Description*	*Subscale*
CU1: Does not care who I hurt to get what I want	Callousness
CU2: Feels bad or guilty when I have done something wrong (r)	Uncaring
CU3: Does not show emotions	Unemotional
CU4: Concerned about the feelings of others (r)	Uncaring
CU5: Does not care if I am in trouble	Callousness
CU6: Does not care about doing things well	Callousness
CU7: Seems very cold and uncaring	Callousness
CU8: Apologizes to persons I have hurt (r)	Uncaring
CU9: Tries not to hurt others’ feelings (r)	Uncaring
CU10: Shows no remorse when I have done something wrong	Callousness
CU11: Feelings of others are unimportant	Callousness
CU12: Does things to make others feel good (r)	Uncaring

*r* reverse coded items

#### Positive and Negative Affect

Positive (5 items; e.g., *active*, *attentive*) and negative (5 items; e.g., *ashamed*, *nervous*) affect were assessed daily (i.e., “indicate to what extent you have felt the way as described by each of the following words today”) with the short-form Positive and Negative Affect Schedule (PANAS-SF; Cooke et al., 2022; Thompson, 2007) on a 5-point Likert scale (1 = *very slightly or not at all* 2 = *a little*, 3 = *moderately*, 4 = *quite a bit*, 5 = *extremely*). MLCFA model fit for the 2-factor model are as follows: CFI = 0.91, TLI = 0.88, RMSEA = 0.04, SRMR_w_ = 0.13, SRMR_b_ = 0.14. The estimated averaged standardized factor loading for the positive affect factor was 0.58 and 0.71 at within- and between-levels, and for the negative affect factor was 0.70 and 0.90 at within- and between-levels. Items were averaged within days with higher scores indicative of higher levels of positive (ω_w_ = 0.63, ω_b_ = 0.88, intra-class correlation [ICC] = 0.71) and negative (ω_w_ = 0.74, ω_b_ = 0.94, ICC = 0.61) affect.

#### Emotional and Conduct Problems

Participants reported their daily emotional and conduct problems using 5 items each from the emotional problems (e.g., *I worry a lot*) and conduct problems (e.g., *I take things that are not mine from home, school, or elsewhere*) subscales of the Strengths and Difficulties Questionnaire (Goodman et al., 1998). Items were rated on a 3-point Likert scale (0 = *not true*, 1 = *somewhat true*, 2 = *certainly true*) and averaged within days. MLCFA model fit for the 2-factor model are as follows: CFI = 0.98, TLI = 0.98, RMSEA = 0.01, SRMR_w_ = 0.10, SRMR_b_ = 0.05. The estimated averaged standardized factor loading for the emotional problems factor was 0.57 and 0.88 at within- and between-levels, and for the conduct problems factor was 0.48 and 0.84 at within- and between-levels. Higher scores represented more emotional (ordinal ω_w_ = 0.70, ordinal ω_b_ = 0.94, ICC = 0.76) and conduct (ordinal ω_w_ = 0.63, ordinal ω_b_ = 0.92, ICC = 0.60) problems that day (i.e., “mark how the following items described you on the basis of how things have been for you today”).

### Analytic Approach

**Fig. 1:**
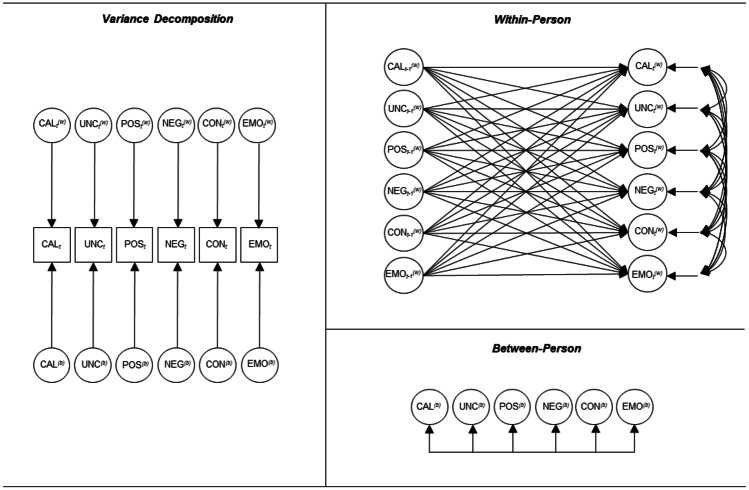
Schematic of DSEM. Note. Observed items are decomposed into time-varying within-level (w) and time-invariant between-level (b) components. CAL = callousness, UNC = uncaring, POS = positive affect, NEG = negative affect, CON = conduct problems, EMO = emotional problems

In M*plus* version 8.6 (Muthén & Muthén, 2021), DSEM (Asparouhov et al., 2018) was conducted to examine both research aims (see Fig. 1 for DSEM schematic of aim 2). Combining time-series and structural equation modeling, this multilevel modeling approach tests temporal relations between measured variables by decomposing the data into within-person (Level 1) and between-person (Level 2) components (Asparouhov et al., 2018; Hamaker et al., 2018). At the within-person level, *inertia*, otherwise known as autoregression or carryover, is an estimate of the extent to which scores return to equilibrium (or persist) over measurement occasions, with higher scores indicating greater resistance to change (Kuppens et al., 2010). Regarding cross-lagged associations, *augmentation* describes positive cross-lagged associations, such that an increase in the level of one construct predicts an increase in another construct at a successive time point (Pe & Kuppens, 2012). By contrast, *blunting* characterizes negative cross-lagged associations, in which an increase in the level of one construct predicts a decrease in another construct at the next time point. In other words, these within-person components assess whether participants’ level at time *t* were significantly predicted by their level of the same or different construct, respectively, at time *t*-1. Contemporaneously, *covariation* specifies whether constructs at the same time point are associated with one another (Krone et al., 2018). Accordingly, at the within-person level (person-level centered), both autoregressive and cross-lagged parameters are estimated. Covariances among residuals between constructs within the same day after controlling for the previous day’s effect (i.e., autoregression and cross-lagged effects) were also estimated. At the between-person level, DSEM models means and (co)variances to examine between-person differences aggregated over the 30-day survey period (i.e., random intercepts). Bayesian Markov Chain Monte Carlo (MCMC) is used for estimation resulting in an entire distribution of possible values for each unknown parameter. Models were estimated using default priors and convergence was established by examining several diagnostic criteria including the Potential Scale Reduction (PSR) statistic, autocorrelation plots, and trace plots. Significance is determined by whether the 95% credible interval (CI) contains value 0 for each parameter. M*plus* code and output are supplied on the Open Science Framework: https://osf.io/n2rdf/?view_only=357ec650bfac4a63905e7ed34efd9255.

## Results

### Callous-unemotional Traits at the Item Level

Descriptive statistics, ICCs, and within-level and between-level CU item correlations are shown in Supplementary Table S1. ICC is the proportion of the total variance that is accounted for by stable or trait-like between-person differences plus measurement error (Hamaker et al., 2018). Accordingly, lower ICCs would indicate more within-person fluctuations. At the within-level, almost all items were significantly positively correlated with each other. At the between-level, items 2 (*feels bad or guilty when I have done something wrong*) and 3 (*does not show emotions*) showed few significant associations with other items and the highest ICC values (0.77 and 0.78, respectively).

After controlling for the previous day’s autoregression and cross-lagged effects, item-level within-day residual correlations are shown in Table 2. Similar to the within-level item correlations presented above, the majority of items were still positively correlated with each other (although magnitudes were smaller) after controlling for the previous day’s effect.

**Table 2:** Within-Person Same Day Residual Correlations and Between-Person Correlations for Callous-Unemotional Traits Items

**Item**	**CU1**	**CU2**	**CU3**	**CU4**	**CU5**	**CU6**	**CU7**	**CU8**	**CU9**	**CU10**	**CU11**	**CU12**
CU1	–	0.01	0.03	0.13^***^	0.14^***^	0.21^***^	0.12^***^	0.08^***^	0.09^***^	0.10^***^	0.07^***^	0.12^***^
CU2	0.22^*^	–	-0.08^***^	0.24^***^	0.12^***^	0.04	-0.04^*^	0.17^***^	0.18^***^	0.02	0.06^**^	0.15^***^
CU3	-0.01	0.46^***^	–	-0.20^***^	0.04^*^	0.05^*^	0.10^***^	-0.12^***^	-0.11^***^	0.04^*^	0.03	-0.06^**^
CU4	0.52^***^	0.23^*^	-0.28^*^	–	0.07^**^	0.07^**^	0.08^***^	0.37^***^	0.38^***^	0.04	0.10^***^	0.34^***^
CU5	0.75^***^	0.17	-0.04	0.57^***^	–	0.31^***^	0.09^***^	0.04^*^	0.06^**^	0.09^***^	0.07^**^	0.05^*^
CU6	0.73^***^	0.13	0.03	0.49^***^	0.87^***^	–	0.19^***^	0.07^**^	0.05^*^	0.11^***^	0.10^***^	0.09^***^
CU7	0.35^**^	0.09	0.34^**^	0.40^**^	0.42^***^	0.61^***^	–	0.05^*^	0.07^**^	0.11^***^	0.09^***^	0.10^***^
CU8	0.50^***^	0.18	-0.23^*^	0.84^***^	0.56^***^	0.48^***^	0.34^**^	–	0.54^***^	0.07^**^	0.08^***^	0.34^***^
CU9	0.53^***^	0.28^*^	-0.19	0.93^***^	0.56^***^	0.51^***^	0.40^***^	0.82^***^	–	0.00	0.08^***^	0.34^***^
CU10	0.67^***^	0.19	-0.05	0.61^***^	0.72^***^	0.75^***^	0.48^***^	0.48^***^	0.62^***^	–	0.11^***^	0.05^*^
CU11	0.76^***^	0.12	0.01	0.60^***^	0.64^***^	0.73^***^	0.54^***^	0.47^***^	0.54^***^	0.81^***^	–	0.08^**^
CU12	0.39^**^	0.15	-0.29^**^	0.90^***^	0.44^***^	0.41^***^	0.36^**^	0.79^***^	0.84^***^	0.49^***^	0.40^***^	–

***M***	0.12	1.00	1.50	0.53	0.29	0.24	0.42	0.63	0.51	0.25	0.17	0.66
***σ***^**2**^	0.09	1.19	1.26	0.37	0.26	0.21	0.36	0.46	0.38	0.17	0.13	0.52

Correlations estimated using the Bayes estimator. Within-day residual correlations are shown above the diagonal. Between-level correlations, means (*M*), and variances (σ^2^**)** are shown below the diagonal. Items 2, 4, 8, 9, 12 are reverse coded

*p ≤ 0.05, **p ≤ 0.01, ***p ≤ 0.001

**Fig. 2:**
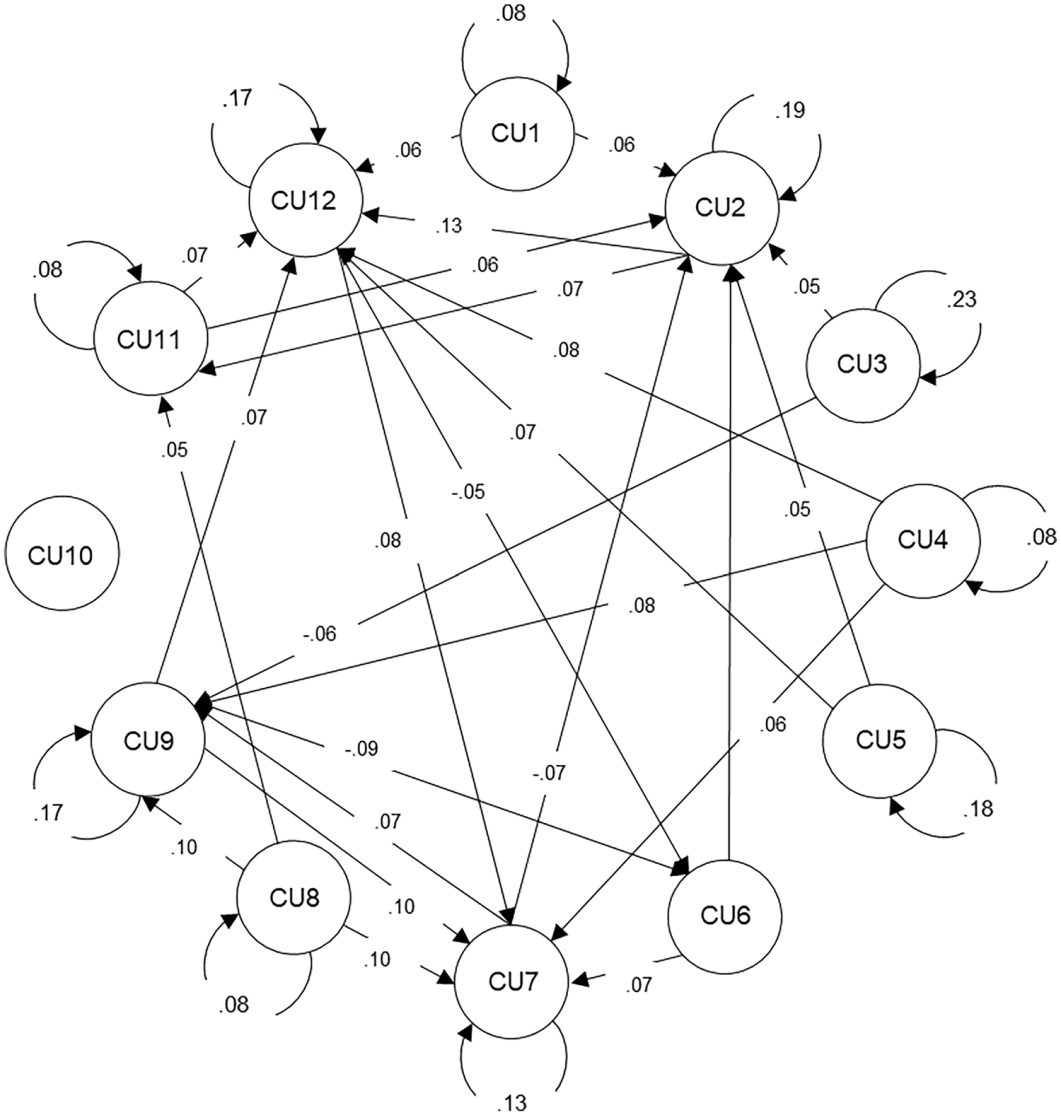
DSEM Standardized Estimates for Callous-Unemotional Traits Items Within-Person Autoregressive (curved arrows) and Cross-Lagged Effects (straight arrows). Note. Only significant effects based on 95% credible intervals are shown. CU = callous-unemotional

Significant item-level autoregressive effects are shown in Fig. 2 (as indicated by curved arrows) and all item-level autoregressive estimates are described in Supplementary Table S2. Except for items 6 (*does not care about doing things well*) and 10 (*shows no remorse when I have done something wrong*), all items showed significant autoregressive effects (βs = 0.08–0.23; 95% CI [0.02–0.18, 0.13–0.28]), suggesting that adolescents experiencing high levels of CU traits on the previous day were more likely to experience high levels the next day compared to their average level. More specifically, items 1 (*does not care who I hurt to get what I want*; β = 0.08; 95% CI [0.02, 0.14]), 4 (*concerned about the feelings of others*; β = 0.08; 95% CI [0.02, 0.15]), 8 (*apologizes to persons I have hurt*; β = 0.08; 95% CI [0.02, 0.14]), and 11 (*feelings of others are unimportant*; β = 0.08; 95% CI [0.02, 0.13]) showed the lowest levels of inertia and item 3 (*does not show emotions*; β = 0.23; 95% CI [0.18, 0.28]) revealed the largest inertia.

Significant item-level cross-lagged effects are shown in Fig. 2 (as indicated by straight arrows) and all item-level cross-lagged estimates are described in Supplementary Table S3. All items, except for item 10, demonstrated cross-lagged effects with at least one other item. However, it is notable that most cross-lagged associations involved only four items as outcomes: items 2 (*feels bad or guilty when I have done something wrong*; βs = -0.07–0.06 95% CIs [-0.12–0.01, -0.02–0.12]), 7 (*seems very cold and uncaring;* βs = 0.06–0.10; 95% CIs [0.01–0.05, 0.11–0.14]), 9 (*tries not to hurt others’ feelings*; βs = -0.06–0.10; 95% CIs [-0.12–0.05, -0.00–0.14]), and 12 (*does things to make others feel good*; βs = 0.06–0.13; 95% CIs [0.00–0.07, 0.12–0.18]). These findings highlight several CU traits that adolescents reported as endorsing higher levels the next day compared to their average level. In addition, most items only showed two significant cross-lagged associations, with items 4, 8, and 9 displaying three cross-lagged effects. All significant cross-lagged associations were augmented, apart from four effects (i.e., 3 → 9, 7 → 2, 9 → 6, and 12 → 6), which revealed blunting effects.

Between-level item correlations, means, and variances are shown in Table 2. Overall, consistent with the between-level descriptive statistics, all items displayed significant positive associations with almost all other items, except items 2 (*feels bad or guilty when I have done something wrong*) and 3 (*does not show emotions*), which showed few significant associations.

### Callous-unemotional Traits and Associations with Subscales

Descriptive statistics, ICCs, and within-level and between-level subscale correlations are shown in Supplementary Table S4. Notably, at the within-level, callousness was significantly positively associated with all variables except positive affect, whereas uncaring was positively associated with negative affect and conduct problems, and negatively with positive affect. At the between-level, callousness was significantly positively associated with all variables and negatively associated with positive affect; uncaring was positively associated with conduct problems and negatively with positive affect. Also, emotional problems displayed the highest ICC (0.76) and uncaring had the lowest ICC (0.60). Table 3 shows within-day residual correlations after controlling for the previous day’s effects. Findings were similar to the within-level item correlations presented above.

**Table 3:** Within-Person Same Day Residual Correlations and Between-Person Correlations for Callous-Unemotional Traits Subscales, Positive Affect, Negative Affect, Conduct Problems, and Emotional Problems

**Item**	**1**	**2**	**3**	**4**	**5**	**6**
1. Callousness	–	0.17^***^	-0.03	0.21^***^	0.22^***^	0.13^***^
2. Uncaring	0.58^***^	–	-0.10^***^	0.09^***^	0.21^***^	0.03
3. Positive Affect	-0.24^*^	-0.36^**^	–	0.06^**^	-0.06^**^	-0.09^***^
4. Negative Affect	0.52^***^	0.08	-0.16	–	0.28^***^	0.48^***^
5. Conduct Problems	0.72^***^	0.59^***^	-0.29^**^	0.52^***^	–	0.17^***^
6. Emotional Problems	0.41^***^	0.09	-0.38^***^	0.88^***^	0.47^***^	–

***M***	0.25	0.66	2.67	1.61	0.18	0.46
***σ***^**2**^	0.12	0.29	0.74	0.46	0.05	0.24

Correlations estimated using the Bayes estimator. Within-day residual correlations are shown above the diagonal. Between-level correlations, means (*M*), and variances (σ^2^**)** are shown below the diagonal

*p ≤ 0.05, **p ≤ 0.01, ***p ≤ 0.001

**Fig. 3:**
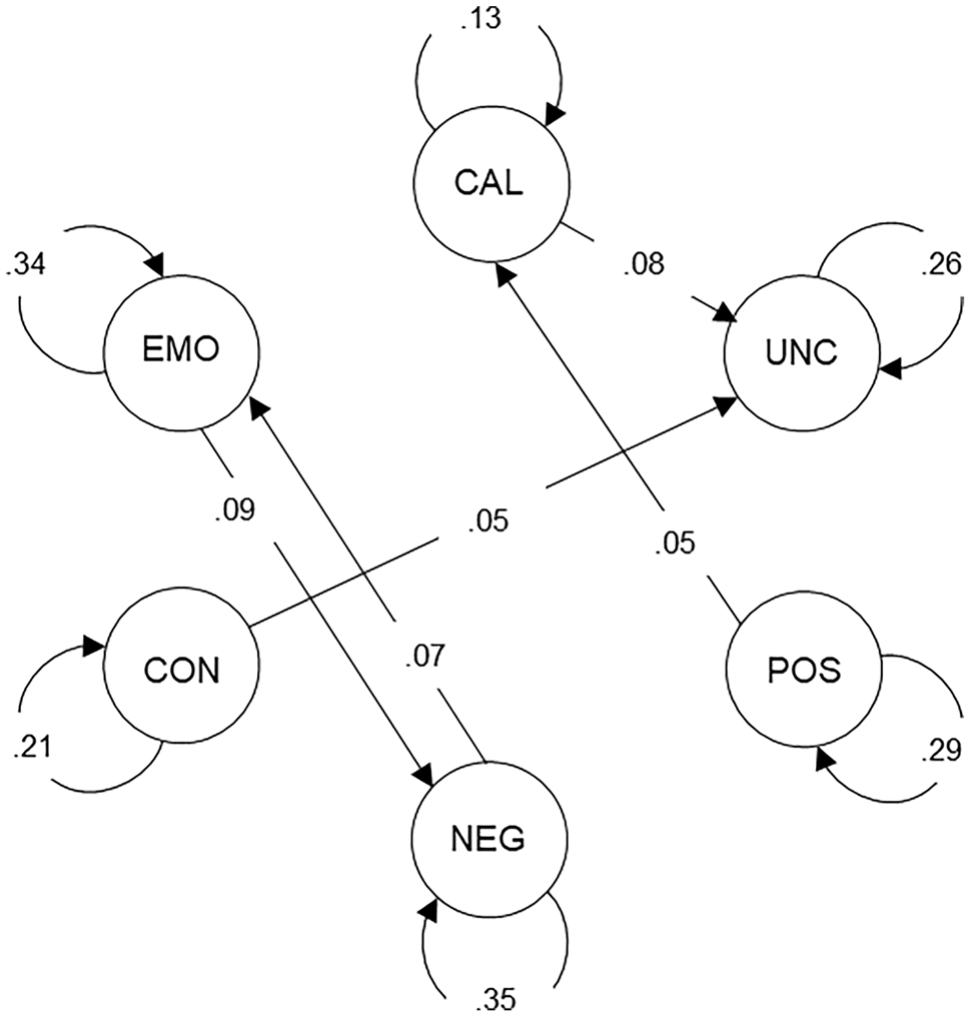
DSEM Standardized Estimates for Callous-Unemotional Traits Subscales, Positive Affect, Negative Affect, Conduct Problems, and Emotional Problems Within-Person Autoregressive (curved arrows) and Cross-Lagged Effects (straight arrows). Note. Only significant effects based on 95% credible intervals are shown. CAL = callousness, UNC = uncaring, POS = positive affect, NEG = negative affect, CON = conduct problems, EMO = emotional problems

Significant autoregressive (curved arrows) and cross-lagged (straight arrows) effects are displayed in Fig. 3 (all estimates are shown in Supplementary Tables S5 and S6, respectively). All variables showed significant autoregressive effects (βs = 0.13–0.35; 95% CIs [0.08–0.29, 0.18–0.41]). Thus, adolescents experiencing high levels of each variable on the previous day were more likely to experience high levels of each variable, respectively, the next day compared to their average level. Regarding cross-lagged effects, all significant associations were positive (i.e., augmentation). Specifically, adolescents who reported higher than their average levels of callousness also reported higher than their average levels of uncaring the next day (β = 0.08; 95% CI [0.03, 0.13]). Similar cross-day associations were observed between conduct problems and uncaring (β = 0.05; 95% CI [0.00, 0.10]), positive affect and callousness (β = 0.05; 95% CI [0.00, 0.10]), negative affect and emotional problems (β = 0.07; 95% CI [0.01, 0.13]), and, finally, emotional problems and negative affect (β = 0.09; 95% CI [0.03, 0.15]). Notably, uncaring did not show any significant cross-lagged effects.

Between-level correlations, means, and variances are shown in Table 3. Notably, adolescents with higher levels of callousness, on average, tended to also have higher average levels of uncaring, negative affect, conduct problems, and emotional problems and lower average levels of positive affect over the month. Those with higher average levels of uncaring also had higher average levels of conduct problems and lower average levels of positive affect over the month.

## Discussion

By collecting intensive longitudinal data and applying analytic approaches that elucidate temporal dynamics, we can expand our understanding of CU traits in a more ecologically valid way. Accordingly, this exploratory study examined whether adolescent CU traits manifest fluctuations across a 1-month observation window. Many CU traits items showed within-person autoregressive and cross-lagged links over the timeframe (i.e., participants’ levels at time *t* were significantly predicted by their levels at time *t*-1), and we described these effects using terminology from the affective dynamics literature. We also illustrated how daily CU traits were associated with daily affect and emotional and conduct problems. Overall, we observed several within- and between-person effects that serve as a starting point for further understanding of temporal dynamics and functional accounts of CU traits and related emotional and behavioral functioning.

### Callous-unemotional Traits in Daily Life

In line with recent research calling for lower-order precision level analyses (Goulter et al., 2022; Kotov et al., 2021), we assessed CU traits at the item-level. Almost all items displayed within-person autoregressive effects (inertia). Of those items, two items that typically load onto callousness (*does not care who I hurt to get what I want*, *feelings of others are unimportant*) and two items that represent uncaring (*concerned about the feelings of others*, *apologizes to persons I have hurt*) had lower inertia and ICC scores (i.e., greater within-person fluctuations) relative to other items. Notably, these items all reflect CU traits related to behaviors directed toward others or others’ emotions or feelings. These items demonstrate greater variability likely because they tap interpersonal interactions with others, which could be less salient and stable relative to items regarding one’s own motivations and behaviors (e.g., *does not care if I am in trouble*, *does not care about doing things well*). Conceptualizations of CU traits point to deficits in both affiliative capacity and emotional responding, and intensive longitudinal designs may prove especially useful in informing socio-emotional accounts of CU traits.

Conversely, *does not show emotions* showed the largest inertia, ICC (i.e., the least within-person fluctuations), and highest between-level mean. This is a notable finding because this item is the only item retained from the 24-item ICU to the brief 12-item version presumably loading onto the unemotional subscale. Although this 12-item adaptation has been well-validated in child, adolescent, and young adult samples (e.g., Hawes et al., 2014b; Zheng et al., 2021), others have emphasized the importance of unemotional items for understanding the broader CU construct (Ray & Frick, 2020). For example, one meta-analytic study found that although the unemotional subscale was only modestly associated with aggression (*r* = 0.06), it was more highly associated with lower empathy (*r* = -0.22; Cardinale & Marsh, 2020). These findings suggest that the unemotional scale may be more consequential for delineating “trait-like” impoverished emotion and empathy aspects of CU traits (Ray & Frick, 2020). Furthermore, both *does not care about doing things well* and *shows no remorse when I have done something wrong* did not show significant autoregressive effects. These results are somewhat expected as it is unlikely that our community sample committed transgressions every day, thus necessitating further research with higher-risk and clinical samples.

A greater understanding of the short-term fluctuations in CU traits can also help inform ethical considerations in this field regarding labelling, stigmatization, and nomenclature. As a construct, CU traits was initially developed by downwardly extending the affective component of adult psychopathy to children and adolescents. Although definitions of psychopathy typically also comprise interpersonal and impulsive/antisocial dimensions, an extensive child and adult literature has revealed that the affective or CU dimension tends to distinguish those individuals with distinct neurocognitive correlates, as well as heightened risk for persistent antisocial behavior (Frick et al., 2014). However, there are reasonable concerns regarding the stigmatization of labelling children and adolescents with the term CU “traits” (Prasad & Kimonis, 2018). Several scholars have accordingly advocated for alternative language, such as CU *behaviors*, *features*, or *symptoms*, particularly in younger children when these characteristics may be less stable (e.g., Schuberth et al., 2019; Waller & Hyde, 2017). Others have made clear at the outset that although they use the term CU “traits” in young samples, they are not suggesting that these indicators are immutable (e.g., Fleming et al., 2022). As well-stated by Kaurin et al. (2022, p. 23) “A stricter focus on dysregulated interpersonal and affective processes in daily life [therefore] may also reform the professional and public view on personality pathology, which is dominated by perceptions of destiny rather than manageable risk.” Overall, our item-level findings provide preliminary granular and temporal evidence of within-person “state” fluctuations of CU traits in real-life.

### Daily Callous-unemotional Traits and Emotional and Behavioral Functioning

We also evaluated whether daily CU traits, including callousness and uncaring, were associated with several indicators of emotional and behavioral functioning. Both callousness and conduct problems augmented uncaring over time. One explanation for these findings may relate to how adolescents with elevated callousness appraise certain situations. It may be the case that adolescents high on callousness with a cognitive predisposition underpinned by fearlessness and deficits in emotional processing interpret certain situational features (e.g., negative interactions with parents or peers) in a way that results in higher levels of uncaring. Also noteworthy, uncaring did not predict any other construct. Structural differences in the ICU have been suggested to be a spurious by-product of method variance (Ray & Frick, 2020); however, our findings contribute some evidence pointing to meaningful differences between CU subscales. Taken together, these findings suggest that uncaring may be better conceptualized as an outcome rather than an antecedent, and callousness could represent a critical treatment target.

An interesting finding was that positive affect was linked with callousness. Previous research found that callousness at baseline negatively predicted positive affect assessed six times per day across a 1-week period (Vize et al., 2022); however, callousness was assessed with a personality measure only at baseline. One explanation for our findings may be due to the specific measure of positive affect assessing high valence constructs, including being active, inspired, and determined (Cooke et al., 2022; Thompson, 2007). It may be the case that callousness also designates resolute individuals characterized by ambition. This notion goes against the DSM-5 “with Limited Prosocial Emotions” criterion specifying an absence of interest in school or work performance (American Psychiatric Association, 2013). However, the current uncaring subscale from the 12-item ICU is operationalized as a lack of concern for others (not activities). In addition, echoing Vize et al. (2022), such traits may be strongly associated with extroversion, which is linked to heightened positive affect (Watson et al., 2015)–although these findings are derived from adult samples. Future studies should consider a broader range of positive affect items to replicate and extend current findings, as well as further research on personality in adolescent samples.

### Strengths and Limitations

Notable strengths of the present study include the naturalistic 30-day diary design, which advances understanding of CU traits under higher ecological validity relative to traditional longitudinal studies. In addition, we applied an analytic approach that disentangles within-person fluctuations from between-person differences. We also evaluated whether daily CU traits were associated with multiple forms of daily emotional and behavioral functioning. However, current findings should be interpreted within the context of some limitations. First, the current sample was primarily comprised of community adolescents from families with higher annual income than the provincial median income. Participants also endorsed lower levels of CU traits (*M* = 6.88; *SD* = 4.26) relative to other studies using the 12-item ICU scale. For example, Colins et al. (2016) found higher levels of CU traits in a sample of justice-involved adolescent girls (*M* = 9.14; *SD* = 5.88). Thus, our results may not generalize to other populations, and as highlighted, it would be particularly important to examine daily CU traits in higher-risk or clinical samples.

Second, all data are solely based on adolescent self-reports–we did not collect data from other informants (e.g., parents) on adolescent daily CU traits. Past research has revealed distinct associations between CU traits and conduct problems across adolescent- and parent-reports (e.g., Goulter & Moretti, 2021). Meta-analytic work has also shown less discrepancies in cross-informant correspondence when informants are reporting on observable behaviors (e.g., externalizing vs. internalizing) or when the behaviors are measured within the same context (De Los Reyes et al., 2015). Regarding CU traits, it may be more difficult for other informants to report on CU traits given these traits are not necessarily readily observable. 

Third, our sample was relatively small, although simulation studies have shown that sufficient power can be achieved for DSEM with a sample size of 100 and a minimum of 25 measured occasions (e.g., Schultzberg & Muthén, 2018). In particular, power at Level 2 is relatively small compared to conventional longitudinal studies, and thus, these between-person effects should be interpreted with caution. However, with over 2,000 observations at the daily level, power at Level 1 is sufficient for the current approach. Because of our sample size, we were not able to optimally investigate gender differences–an important direction for future research given most studies examining CU traits have relied predominantly on male samples.

### Future Directions and Implications

By decomposing “trait” and “state”-like aspects of CU traits, current findings inform understanding of fluctuations in CU traits and lay a foundation for future intensive longitudinal designs to test critical questions in the field. For instance, adolescence represents a sensitive period marked by distinct change including within neural systems involved in social, emotional, and motivational processes (Dahl et al., 2018). Because of this, studies testing daily dynamics among adolescents may be particularly well-suited to reveal important information regarding socio-behavioral functioning. Atypical levels of CU traits have also been observed in samples as young as preschool age (Kimonis et al., 2016), and developmental models describe early childhood as the period in which the emergence of conscience and empathy typically occurs (Kochanska, 1993). Thus, future research should also investigate daily CU traits in younger samples. Ideally, CU traits would be examined at multiple developmental stages across multiple timescales, and measurement burst designs that combine micro and macro longitudinal methods could identify changes in short-term CU dynamics and their relations with long-term outcomes. Future research should also apply multiple measurement approaches (e.g., self-report, observational, psychophysiological) to better characterize the emotional components of daily experiences–a particularly important avenue in CU traits research given the distinct psychophysiological profiles among these samples (Fanti et al., 2019).

It will also be crucial to investigate the role of specific situational features in CU traits fluctuations and employ different intensive longitudinal methods to test these relations. For example, the present study employed a time-based sampling scheme prompting participants at 5 pm each day. By contrast, other research may use a variable-interval scheme generating prompts at random throughout the day to further enhance ecological validity (Russell & Gajos, 2020). Future studies may also want to consider event contingent designs, which could be useful for examining negative interactions with parents, peers, or the broader environment. Theoretical and empirical works highlight the role of warm and supportive parent–child relationships in promoting empathy development and eliciting positive affect (Kochanska, 1993). Intensive longitudinal methods with parent–child dyads (e.g., Xu & Zheng, 2022) have the potential to further advance understanding of familial relations in the development of CU traits. Particularly, parenting behaviors, including parental warmth and harsh discipline, have been linked to child CU traits (Goulter et al., 2020; Waller et al., 2013; Zheng et al., 2017), and recent daily diary studies have demonstrated substantial daily fluctuations in parental warmth (Xu & Zheng, 2023). Thus, examining how daily parenting behaviors are associated with CU traits may provide modifiable targets in daily family processes for future treatment efforts. In adolescence, research may want to examine the role of peer affiliation and interactions in daily CU traits fluctuations. Although the present study assessed negative affect, a greater understanding of the dynamics of stress and associated stressful experiences would also be particularly informative.

## Conclusion

In this exploratory study, we observed meaningful fluctuations of CU traits at both item- and subscale-levels. These findings suggest that CU traits may be more susceptible to change and malleable than previously thought. In addition, callousness and uncaring subscales demonstrated distinct cross-day dynamics in relation to emotional and behavioral problems. Here, we have laid a foundation for future research focus applying intensive longitudinal methods to advance understanding of CU traits in daily life. Further functional information gained from these methods can potentially inform intervention efforts by offering modifiable targets situated in daily lives to promote personalized and just-in-time interventions. By conducting sampling in real-time in real-world situations, we can further inform personalized models of treatment targeting daily processes.

### Supplementary Information

Below is the link to the electronic supplementary material.

Supplementary file1 (PDF 306 KB)

## Data Availability

Data are available by emailing the last author. Code and output are available on the Open Science Framework: https://osf.io/n2rdf/?view_only=357ec650bfac4a63905e7ed34efd9255.
