# Supplementary material for: Callous-Unemotional Traits in Adolescents' Daily Life: Associations with Affect and Emotional and Conduct Problems
Source: Res Child Adolesc Psychopathol. 2023 May 30;52(1):51–63. doi: 10.1007/s10802-023-01077-6 (PMC10787886; doi:10.1007/s10802-023-01077-6)
Supplement: Supplementary file 1 — Supplementary file1 (PDF 306 KB) [file 10802_2023_1077_MOESM1_ESM.pdf]

Callous-Unemotional Traits in Adolescents' Daily Life: Associations with Affect and Emotional and Conduct Problems

Natalie Goulter
School of Psychology, Newcastle University, United Kingdom
Department of Psychology, Simon Fraser University, Canada
Eric M. Cooke
Criminal Justice Program, Bowling Green State University, United States of America
Yao Zheng
Department of Psychology, University of Alberta, Canada

Author Note

Natalie Goulter https://orcid.org/0000-0003-0682-7781
Eric M. Cooke https://orcid.org/0000-0002-5630-3217
Yao Zheng https://orcid.org/0000-0002-0302-0598

Corresponding Author Contact Details

Natalie Goulter, natalie.goulter@newcastle.ac.uk

Supplementary Materials

Supplementary Table S1

Means, Standard Deviations, ICCs, and Correlations for Callous-Unemotional Traits Items

Item	*M*	*SD*	*Range*	ICC	CU1	CU2	CU3	CU4	CU5	CU6	CU7	CU8	CU9	CU10	CU11	CU12
CU1	.11	.38	0–3	.66	—	.03	.03	.13***	.15***	.21***	.11***	.08***	.09***	.10***	.08***	.12***
CU2	1.02	1.16	0–3	.77	.22	—	-.07***	.25***	.14***	.05*	-.04	.18***	.19***	.01	.07***	.17***
CU3	1.52	1.12	0–3	.78	-.01	.45***	—	-.20***	.05*	.05*	.09***	-.13***	-.11***	.04*	.03	-.06***
CU4	.52	.74	0–3	.60	.51***	.21	-.28	—	.08***	.08***	.09***	.39***	.40***	.04*	.10***	.34***
CU5	.28	.60	0–3	.71	.75***	.18	-.02	.58***	—	.31***	.09***	.04***	.06***	.09***	.08***	.06***
CU6	.24	.56	0–3	.70	.73***	.14	.04	.50***	.86***	—	.19***	.08***	.06***	.12***	.10***	.10***
CU7	.43	.73	0–3	.66	.35***	.06	.33***	.40***	.40***	.61***	—	.06***	.09***	.11***	.09***	.11***
CU8	.63	.83	0–3	.62	.50***	.18	-.25	.83***	.55***	.48***	.33***	—	.56***	.07***	.09***	.36***
CU9	.50	.78	0–3	.62	.54***	.28	-.19	.92***	.55***	.52***	.40***	.82***	—	.01	.10***	.36***
CU10	.24	.58	0–3	.61	.67***	.19	-.03	.61***	.71***	.73***	.47***	.47***	.61***	—	.12***	.05*
CU11	.16	.50	0–3	.56	.75***	.11	-.01	.60***	.62***	.71***	.55***	.47***	.54***	.80***	—	.09***
CU12	.66	.82	0–3	.68	.39***	.13	-.29***	.89***	.44***	.41***	.37***	.78***	.83***	.47***	.40***	—

Note: Item correlations estimated using the Bayes estimator. Within-level item correlations are shown above the diagonal. Between-level item correlations are shown below the diagonal. Items 2, 4, 8, 9, 12 are reverse coded. *M* = mean, *SD* = standard deviation, ICC = intraclass correlation coefficient. $^{*}p \leq .05$, $^{**}p \leq .01$, $^{***}p \leq .001$

Supplementary Table S2

DSEM Standardized Estimates for Callous-Unemotional Traits Items Within-Person Autoregressive Effects

**Item**	**β**	***SD***	***p***	**95% CI_{lower}**	**95% CI_{upper}**
Within-Person Autoregressive					
CU1 → CU1	**.08**	**.03**	**.001**	**.03**	**.14**
CU2 → CU2	**.19**	**.03**	**<.001**	**.14**	**.24**
CU3 → CU3	**.23**	**.03**	**<.001**	**.18**	**.28**
CU4 → CU4	**.08**	**.03**	**.004**	**.02**	**.15**
CU5 → CU5	**.18**	**.03**	**<.001**	**.12**	**.23**
CU6 → CU6	.05	.03	.043	-.01	.11
CU7 → CU7	**.13**	**.03**	**<.001**	**.08**	**.18**
CU8 → CU8	**.08**	**.03**	**.004**	**.02**	**.14**
CU9 → CU9	**.17**	**.03**	**<.001**	**.10**	**.23**
CU10 → CU10	.05	.03	.034	-.00	.10
CU11 → CU11	**.08**	**.03**	**.002**	**.02**	**.13**
CU12 → CU12	**.17**	**.03**	**<.001**	**.11**	**.22**

Note: Bolded indicates significance based on CI. Items 2, 4, 8, 9, 12 are reverse coded. β = standardized beta, *SD* = standard deviation, CI = credible interval.

Supplementary Table S3

DSEM Standardized Estimates for Callous-Unemotional Traits Items Within-Person Cross-Lagged Effects

Item	β	*SD*	*p*	95% CI_{lower}	95% CI_{upper}
Within-Person Cross-Lagged					
CU1					
CU1 → CU2	**.06**	**.03**	**.010**	**.01**	**.11**
CU1 → CU3	.00	.03	.498	-.05	.05
CU1 → CU4	-.04	.03	.075	-.10	.02
CU1 → CU5	.04	.03	.077	-.02	.09
CU1 → CU6	.04	.03	.109	-.02	.09
CU1 → CU7	-.01	.03	.378	-.06	.04
CU1 → CU8	-.01	.03	.410	-.07	.06
CU1 → CU9	.00	.03	.482	-.06	.07
CU1 → CU10	.02	.03	.247	-.04	.07
CU1 → CU11	-.03	.03	.120	-.08	.02
CU1 → CU12	**.06**	**.03**	**.022**	**.00**	**.12**
CU2					
CU2 → CU1	-.02	.03	.180	-.07	.03
CU2 → CU3	.03	.02	.153	-.02	.07
CU2 → CU4	-.04	.03	.081	-.10	.02
CU2 → CU5	-.01	.03	.350	-.06	.04
CU2 → CU6	.02	.03	.228	-.03	.07
CU2 → CU7	-.02	.02	.242	-.06	.03
CU2 → CU8	-.03	.03	.184	-.09	.03
CU2 → CU9	.04	.03	.122	-.02	.10
CU2 → CU10	-.01	.02	.297	-.06	.04
CU2 → CU11	**.07**	**.03**	**.003**	**.02**	**.12**
CU2 → CU12	**.13**	**.03**	**<.001**	**.07**	**.18**
CU3					
CU3 → CU1	.01	.03	.331	-.04	.06
CU3 → CU2	**.05**	**.03**	**.016**	**.00**	**.10**
CU3 → CU4	.01	.03	.366	-.05	.07
CU3 → CU5	.03	.03	.158	-.03	.08
CU3 → CU6	-.04	.03	.099	-.09	.02
CU3 → CU7	.00	.02	.439	-.04	.05
CU3 → CU8	.01	.03	.363	-.05	.05
CU3 → CU9	**-.06**	**.03**	**.023**	**-.12**	**-.00**
CU3 → CU10	.01	.02	.416	-.04	.05
CU3 → CU11	-.01	.03	.385	-.06	.04
CU3 → CU12	.01	.03	.396	-.05	.06
CU4					
CU4 → CU1	-.03	.03	.115	-.09	.02
CU4 → CU2	-.01	.03	.432	-.06	.05
CU4 → CU3	-.00	.03	.460	-.05	.05
CU4 → CU5	.02	.03	.194	-.03	.08
CU4 → CU6	-.01	.03	.354	-.07	.05
CU4 → CU7	**.06**	**.03**	**.009**	**.01**	**.11**
CU4 → CU8	.01	.03	.440	-.06	.07
CU4 → CU9	**.08**	**.03**	**.012**	**.01**	**.14**
CU4 → CU10	.02	.03	.232	-.03	.07
CU4 → CU11	.00	.03	.491	-.05	.05
CU4 → CU12	**.08**	**.03**	**.002**	**.03**	**.14**

CU5					
CU5 → CU1	.05	.03	.046	-.01	.10
CU5 → CU2	**.05**	**.03**	**.021**	**.00**	**.10**
CU5 → CU3	.03	.03	.102	-.02	.08
CU5 → CU4	-.05	.03	.045	-.11	.01
CU5 → CU6	-.05	.03	.042	-.10	.01
CU5 → CU7	-.01	.03	.422	-.05	.04
CU5 → CU8	.02	.03	.273	-.04	.08
CU5 → CU9	.00	.03	.470	-.06	.07
CU5 → CU10	.02	.03	.196	-.03	.07
CU5 → CU11	-.01	.03	.310	-.06	.04
CU5 → CU12	**.07**	**.03**	**.016**	**.00**	**.12**
CU6					
CU6 → CU1	.04	.03	.101	-.02	.09
CU6 → CU2	**.06**	**.03**	**.017**	**.00**	**.11**
CU6 → CU3	.00	.03	.479	-.05	.05
CU6 → CU4	.00	.03	.456	-.05	.06
CU6 → CU5	.02	.03	.235	-.03	.08
CU6 → CU7	**.07**	**.03**	**.004**	**.02**	**.12**
CU6 → CU8	.01	.03	.419	-.06	.07
CU6 → CU9	-.03	.03	.186	-.09	.03
CU6 → CU10	-.01	.03	.379	-.06	.04
CU6 → CU11	-.01	.03	.337	-.06	.04
CU6 → CU12	.03	.03	.157	-.03	.09
CU7					
CU7 → CU1	.01	.03	.311	-.04	.06
CU7 → CU2	**-.07**	**.03**	**.002**	**-.12**	**-.02**
CU7 → CU3	.02	.03	.189	-.03	.07
CU7 → CU4	-.02	.03	.198	-.08	.03
CU7 → CU5	.00	.03	.455	-.05	.06
CU7 → CU6	-.03	.03	.133	-.08	.02
CU7 → CU8	.06	.03	.030	-.00	.12
CU7 → CU9	**.07**	**.03**	**.011**	**.01**	**.13**
CU7 → CU10	-.02	.03	.174	-.07	.03
CU7 → CU11	-.02	.03	.229	-.07	.03
CU7 → CU12	.02	.03	.255	-.04	.08
CU8					
CU8 → CU1	-.04	.03	.072	-.09	.01
CU8 → CU2	.02	.03	.197	-.03	.07
CU8 → CU3	-.02	.03	.170	-.07	.03
CU8 → CU4	.04	.03	.103	-.02	.09
CU8 → CU5	-.02	.03	.191	-.08	.03
CU8 → CU6	-.04	.03	.075	-.09	.02
CU8 → CU7	**.10**	**.02**	**<.001**	**.05**	**.14**
CU8 → CU9	**.10**	**.03**	**.001**	**.04**	**.16**
CU8 → CU10	.02	.03	.184	-.03	.07
CU8 → CU11	**.05**	**03**	**.020**	**.00**	**.10**
CU8 → CU12	.05	.03	.038	-.01	.11
CU9					
CU9 → CU1	.02	.03	.258	-.03	.07
CU9 → CU2	.01	.03	.291	-.04	.06
CU9 → CU3	-.02	.03	.258	-.07	.03
CU9 → CU4	.03	.03	.186	-.03	.08
CU9 → CU5	-.01	.03	.397	-.06	.05
CU9 → CU6	**-.09**	**.03**	**<.001**	**-.14**	**-.04**
CU9 → CU7	**.10**	**.02**	**<.001**	**.05**	**.14**

CU9 → CU8	.06	.03	.003	-.00	.11
CU9 → CU10	-.02	.02	.245	-.06	.03
CU9 → CU11	.04	.03	.064	-.01	.09
CU9 → CU12	**.07**	**.03**	**.009**	**.01**	**.12**
CU10					
CU10 → CU1	.03	.03	.111	-.02	.09
CU10 → CU2	-.01	.03	.328	-.07	.04
CU10 → CU3	.02	.03	.209	-.03	.07
CU10 → CU4	-.01	.03	.414	-.07	.05
CU10 → CU5	-.00	.03	.470	-.06	.05
CU10 → CU6	-.03	.03	.129	-.09	.02
CU10 → CU7	.05	.03	.035	-.00	.10
CU10 → CU8	-.00	.03	.482	-.07	.06
CU10 → CU9	.02	.03	.295	-.05	.08
CU10 → CU11	.02	.03	.265	-.04	.07
CU10 → CU12	.02	.03	.295	-.05	.08
CU11					
CU11 → CU1	.05	.03	.027	-.00	.11
CU11 → CU2	**.06**	**.03**	**.010**	**.01**	**.12**
CU11 → CU3	.02	.03	.219	-.03	.07
CU11 → CU4	-.05	.03	.048	-.11	.01
CU11 → CU5	.04	.03	.077	-.01	.09
CU11 → CU6	-.02	.03	.226	-.08	.03
CU11 → CU7	.02	.03	.190	-.03	.07
CU11 → CU8	-.02	.03	.304	-.08	.05
CU11 → CU9	.02	.03	.237	-.04	.09
CU11 → CU10	.02	.03	.236	-.04	.07
CU11 → CU12	**.07**	**.03**	**.010**	**.01**	**.13**
CU12					
CU12 → CU1	.03	.03	.178	-.03	.08
CU12 → CU2	.03	.03	.110	-.02	.09
CU12 → CU3	.00	.03	.440	-.05	.05
CU12 → CU4	-.01	.03	.438	-.06	.06
CU12 → CU5	-.00	.03	.490	-.05	.06
CU12 → CU6	**-.05**	**.03**	**.021**	**-.11**	**-.00**
CU12 → CU7	**.08**	**.03**	**.001**	**.03**	**.13**
CU12 → CU8	-.02	.03	.227	-.09	.04
CU12 → CU9	.03	.03	.144	-.03	.10
CU12 → CU10	.01	.03	.426	-.04	.05
CU12 → CU11	.04	.03	.060	-.01	.09

Note: Bolded indicates significance based on CI. Items 2, 4, 8, 9, 12 are reverse coded. β = standardized beta, *SD* = standard deviation, CI = credible interval.

Supplementary Table S4

Means, Standard Deviations, ICCs, and Correlations for CU Traits, Positive Affect, Negative Affect, Conduct Problems, and Emotional Problems Subscales

**Item**	***M***	***SD***	***Range***	**ICC**	**1**	**2**	**3**	**4**	**5**	**6**
1. Callousness	.24	.37	0.00-2.33	.66	—	.19***	-.02	.20***	.23***	.14**
2. Uncaring	.66	.62	0.00-3.00	.60	.57***	—	-.12***	.10*	.22***	.04
3. Positive Affect	2.66	.94	1.00-5.00	.70	-.24*	-.34***	—	.08	-.05	-.07
4. Negative Affect	1.59	.80	1.00-5.00	.62	.51***	.07	-.16	—	.28***	.51***
5. Conduct Problems	.18	.26	0.00-1.40	.61	.71***	.58***	-.29**	.50***	—	.17***
6. Emotional Problems	.44	.51	0.00-2.00	.76	.40***	.07	-.38***	.87***	.46***	—

Note: Item correlations estimated using the Bayes estimator. Within-level item correlations are shown above the diagonal. Between-level item correlations are shown below the diagonal. *M* = mean, *SD* = standard deviation, ICC = intraclass correlation coefficient. $^{*}p \leq .05$, $^{**}p \leq .01$, $^{***}p \leq .001$

Supplementary Table S5

DSEM Standardized Estimates for Callous-Unemotional Traits, Positive Affect, Negative Affect, Conduct Problems, and Emotional Problems Subscales Within-Person Autoregressive Effects

**Item**	**β**	***SD***	***p***	**95% CI_{lower}**	**95% CI_{upper}**
Within-Person Autoregressive					
Callousness → Callousness	**.13**	**.03**	**<.001**	**.08**	**.18**
Uncaring → Uncaring	**.26**	**.03**	**<.001**	**.21**	**.32**
Positive Affect → Positive Affect	**.29**	**.03**	**<.001**	**.24**	**.34**
Negative Affect → Negative Affect	**.35**	**.03**	**<.001**	**.29**	**.41**
Conduct Problems → Conduct Problems	**.21**	**.03**	**<.001**	**.16**	**.26**
Emotional Problems → Emotional Problems	**.34**	**.03**	**<.001**	**.29**	**.40**

Note: Bolded indicates significance based on CI. β = standardized beta, *SD* = standard deviation, CI = credible interval.

Supplementary Table S6

DSEM Standardized Estimates for Callous-Unemotional Traits, Positive Affect, Negative Affect, Conduct Problems, and Emotional Problems Subscales Within-Person Cross-Lagged Effects

**Item**	**β**	***SD***	***p***	**95% CI_{lower}**	**95% CI_{upper}**
Within-Person					
Cross-Lagged					
Callousness					
Callousness → Uncaring	**.08**	**.03**	**.001**	**.03**	**.13**
Callousness → Positive Affect	.05	.03	.032	-.00	.10
Callousness → Negative Affect	-.04	.03	.082	-.11	.02
Callousness → Conduct Problems	.01	.03	.334	-.04	.06
Callousness → Emotional Problems	.05	.03	.054	-.01	.11
Uncaring					
Uncaring → Callous	.04	.03	.078	-.01	.09
Uncaring → Positive Affect	-.03	.02	.150	-.07	.02
Uncaring → Negative Affect	-.02	.03	.240	-.08	.04
Uncaring → Conduct Problems	.05	.03	.026	.00	.10
Uncaring → Emotional Problems	.00	.03	.456	-.06	.06
Positive Affect					
Positive Affect → Callous	**.05**	**.03**	**.024**	**.00**	**.10**
Positive Affect → Uncaring	-.04	.03	.070	-.09	.01
Positive Affect → Negative Affect	.05	.03	.063	-.01	.10
Positive Affect → Conduct Problems	-.01	.03	.280	-.06	.04
Positive Affect → Emotional Problems	-.00	.03	.452	-.06	.05
Negative Affect					
Negative Affect → Callous	-.00	.03	.439	-.05	.05
Negative Affect → Uncaring	.01	.02	.410	-.05	.05
Negative Affect → Positive Affect	.05	.02	.026	-.00	.09
Negative Affect → Conduct Problems	-.02	.03	.173	-.07	.03
Negative Affect → Emotional Problems	**.07**	**.03**	**.009**	**.01**	**.13**
Conduct Problems					
Conduct Problems → Callous	.03	.03	.153	-.02	.08
Conduct Problems → Uncaring	**.05**	**.03**	**.023**	**.00**	**.10**
Conduct Problems → Positive Affect	.04	.03	.053	-.01	.09
Conduct Problems → Negative Affect	.00	.03	.498	-.06	.06
Conduct Problems → Emotional Problems	.01	.03	.412	-.05	.06
Emotional Problems					
Emotional Problems → Callous	.02	.02	.223	-.03	.07
Emotional Problems → Uncaring	.03	.02	.129	-.02	.08
Emotional Problems → Positive Affect	.03	.02	.084	-.01	.08
Emotional Problems → Negative Affect	**.09**	**.03**	**.001**	**.03**	**.15**
Emotional Problems → Conduct Problems	-.03	.03	.094	-.08	.02

Note: Bolded indicates significance based on CI. β = standardized beta, *SD* = standard deviation, CI = credible interval.
